# ESP-102, a Combined Herbal Extract of* Angelica gigas*,* Saururus chinensis*, and* Schisandra chinensis*, Changes Synaptic Plasticity and Attenuates Scopolamine-Induced Memory Impairment in Rat Hippocampus Tissue

**DOI:** 10.1155/2016/8793095

**Published:** 2016-05-19

**Authors:** Hyun-Bum Kim, Eun-Sang Hwang, Ga-Young Choi, Seok Lee, Tae-Suk Park, Cheol-Won Lee, Eun-Suk Lee, Young-Choong Kim, Sang Seong Kim, Sung-Ok Lee, Ji-Ho Park

**Affiliations:** ^1^Department of East-West Medical Science, Graduate School of East-West Medical Science, Kyung Hee University, Deogyeong-daero, Giheung-gu, Yongin-si, Gyeonggi-do 446-701, Republic of Korea; ^2^Department of East-West Medicine, Graduate School of East-West Medical Science, Kyung Hee University, Deogyeong-daero, Giheung-gu, Yongin 446-701, Republic of Korea; ^3^College of Pharmacy and Research Institute of Pharmaceutical Science, Seoul National University, San 56-1, Sillim-Dong, Gwanak-Gu, Seoul 151-742, Republic of Korea; ^4^College of Pharmacy, Hanyang University, Hanyangdaehak-ro, Sannok-go, Ansan, Gyeonggi-do 15588, Republic of Korea; ^5^Department of Oriental Medicinal Materials and Processing, College of Life Science, Kyung Hee University, Deogyeong-daero, Giheung-gu, Yongin-si, Gyeonggi-do 446-701, Republic of Korea; ^6^Research Institute of Medical Nutrition, Kyung Hee University, Deogyeong-daero, Giheung-gu, Yongin-si, Gyeonggi-do 446-701, Republic of Korea

## Abstract

ESP-102, an extract from* Angelica gigas, Saururus chinensis*, and* Schisandra chinensis*, has been used as herbal medicine and dietary supplement in Korea. Despite the numerous bioactivities* in vitro* and* in vivo* studies, its effects on neuronal networks remain elusive. To address the neuronal effect, we examined synaptic plasticity in organotypic hippocampal slice culture with multielectrode array. Our results showed an increase in excitatory postsynaptic potential (EPSP), indicating the induction of long-term potentiation (LTP), in the presence of ESP-102. In addition, the neuroprotective effect of ESP-102 was also tested by application of scopolamine to the hippocampal slice. Interestingly, ESP-102 competitively antagonized the preventative LTP effect induced by scopolamine. The scopolamine-induced reduction in brain-derived neurotrophic factor (BDNF) and GluR-2 expression was also rescued by ESP-102. In terms of mode of action, ESP-102 appears to act on the presynaptic region independent of AMPA/NMDA receptors. Based on these findings, ESP-102 can be suggested as a novel herbal ingredient with memory enhancing as well as neuroprotective effects.

## 1. Introduction

ESP-102 is a standardized combined extract of* Angelica gigas*,* Saururus chinensis,* and* Schisandra chinensis* [1–3]. In traditional medicine, it is used alone or in combination with other herbs to gain certain effects. In our previous research, we showed that acute or prolonged daily oral treatment of mice with ESP-102 significantly reduced scopolamine-induced memory deficits in both the passive avoidance test and the Morris water maze test [1]. ESP-102 was also demonstrated to have a significant neuroprotective activity against neurotoxicity induced by glutamate in primary cultures of rat cortical cells as a result of its antioxidative property [3]. ESP-102 also showed an ameliorating effect in an A*β* (1–42) peptide-induced memory impairment model [2]. These findings suggest that ESP-102 might have important neuroprotective properties against the neuronal cell death and cognitive impairments often observed in Alzheimer's disease, stroke, ischemic injury, and other neurodegenerative diseases, in addition to its antioxidative and anti-inflammatory effects [1]. Despite its beneficial roles, the direct effect of ESP-102 on learning and memory in a functional neural network is not fully understood. Understanding the long-term potentiation (LTP) phenomenon in the hippocampus has been considered a useful way to elucidate the underlying mechanism of learning and memory functions in the central nervous system [4]. In order to induce LTP, the functional neural network should be in a healthy state and a high frequency of theta-burst stimulation (TBS) is usually applied to the specific network sites [5, 6]. This results in a dramatic change in the working nature of the whole neural network because a strong TBS might not be localized in the vicinity of target but spread out to the whole network [7, 8]. Recently accumulating knowledge about the LTP mechanism has indicated that LTP enhances learning and memory processes in various pathological conditions [9–11]. Improved understanding of synaptic plasticity will help us verify the pharmacological effects of drug candidates and their effects on the neuronal network [9].

In this study, we investigate the role of ESP-102 in neuronal network modulation. LTP induction was observed in organotypic hippocampal slice cultures after ESP-102 treatment. ESP-102 also induced a significant enhancement in synaptic transmission, implicating a neuroprotective effect. The results of this study encourage further study on the effects of natural extracts and their components on central nervous system function.

## 2. Materials and Methods

### 2.1. ESP-102 Preparation and Chemical Treatments

ESP-102 is a standardized combined extract consisting of extracts from* Angelica gigas* roots,* Saururus chinensis* herb, and* Schisandra chinensis* fruits in the ratio of 8 : 1 : 1. This ratio demonstrated the greatest efficacy in our previous studies and its effects were greater than those of* Angelica gigas* alone (data not shown). ESP-102 was suspended in oxygenated (95% O_2_, 5% CO_2_) artificial cerebrospinal fluid (aCSF, containing 114 mM NaCl, 25 mM NaHCO_3_, 25 mM glucose, 3 mM KCl, 2 mM CaCl_2_, 1.3 mM MgCl_2_, and 20 mM HEPES, pH 7.4) at concentrations of 1, 10, and 100 *μ*g/mL for treatment with a flow rate at a speed of 3 mL/min. ESP-102 and donepezil were provided by Daewoong Pharmaceuticals Co. (Seoul, Korea). Decursin in* A. gigas*, sauchinone in* S. chinensis*, and schizandrin in* S. sinensis* were selected for confirmation of consistency among preparations. The average amount of decursin, sauchinone, and schizandrin in ESP-102 was 3.02 ± 1.49%, 0.05 ± 0.03%, and 0.04 ± 0.03%, respectively. For chemical treatment, scopolamine hydrobromide (10 mM; 6533-68-2, Sigma-Aldrich, St. Louis, MO, USA), AP-5 (10 *μ*M; 76316-31-3, Sigma-Aldrich), and CNQX (10 *μ*M; 115066-14-3, Sigma-Aldrich) were suspended in oxygenated aCSF solution.

### 2.2. Preparation of Organotypic Hippocampal Slice Cultures

**Figure 1:**
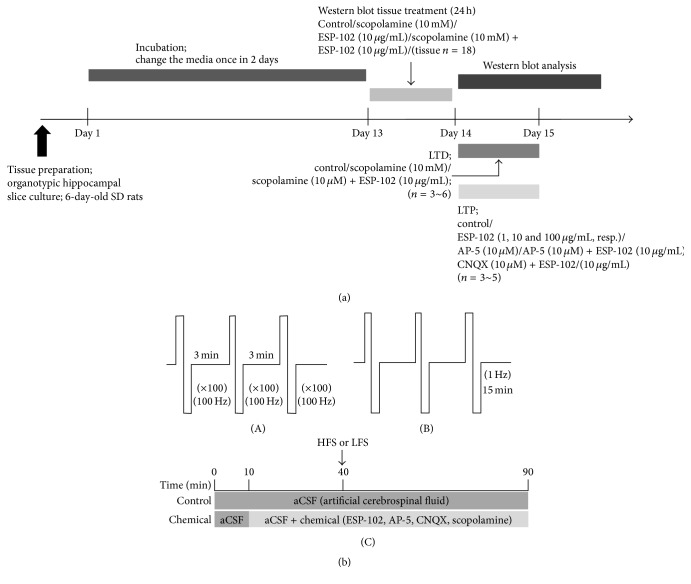
Experiment design and electrophysiology study: stimulation protocol. (a) ESP-102 experimental schedule. (b) (A) High-frequency stimulation (HFS) for long-term potentiation (LTP) induction, (B) low-frequency stimulation (LFS) for long-term depression (LTD) induction, and (C) experimental schedule for LTP and LTD.

The experimental schedule is summarized in Figure 1. All experimental protocols were reviewed and approved by the Institutional Care and Use Committee (KHUASP (SU)-13-03) of Kyung Hee University (Yongin, Republic of Korea). Preparation of organotypic hippocampal slice cultures was based on the interface method [12]. All procedures for culture preparation were carried out in a sterilized room with autoclaved equipment. Seven-day-old Sprague-Dawley rats [12] were decapitated and their brains were quickly removed with scissors and forceps and immediately soaked in ice-cold HBSS-medium (LB 003-01, Sigma-Aldrich) with 20 mM HEPES (H-4034, Sigma-Aldrich). The frontal cortex and the cerebellum were delicately removed and the hippocampus was isolated. These tissues were cut into 350 *μ*m slices using a tissue chopper (Mickle Laboratory Engineering Co., Surrey, UK). Each slice was placed on a 4.0 *μ*m polytetrafluorethylene membrane insert (Millicell-CM; Millipore Co., Bedford, MA, USA), which was set into a well of a 6-well plate (32006, STL Life Science, Anyang-si, Gyeonggi-do, Republic of Korea) filled with 1 mL of culture medium composed of 50% Minimum Essential Medium (LM 007-01, JBI, Daegu, Republic of Korea), 25% horse serum (S 104-01, JBI), 25% Hank's balanced salt solution (LB 003-1, JBI), 6 g/L D-glucose (G-7528, Sigma), 1 mM L-glutamine (G-8540, Sigma), 20 mM HEPES (H-4034, Sigma), and 1% penicillin-streptomycin (LS 202-02, Gibco BRL, USA). The solution was titrated to pH 7.1 with NaOH and HCl solutions. The medium was replaced every other day and the culture slices were incubated for 14 days at 36°C in 5% CO_2_ with 95% humidity.

### 2.3. Preparation of Hippocampal Slice Tissue on the Microelectrode Array Probes

The microelectrode array (MEA; Multi Channel Systems GmbH, Karlsruhe, Germany) consisted of an 8 × 8 array of microelectrodes (diameter 30 *μ*m; interelectrode distance 200 *μ*m) with porous titanium nitride (TiN) as a topcoat to minimize impedance. The MEA surface was rinsed with distilled water to detach tissue residues, bathed for 1 h in 2% ultrasonol 7 (Carl Roth GmbH), and lightly cleaned with a brush. After coating with 0.1% polyethylenimine (PEI; Sigma), the MEA was air-dried on a clean bench and sterilized with UV light for at least 3 h. The probes were rinsed 2-3 times with sterile distilled water before each use. For each experiment a single representative hippocampal slice (generally the third or fourth one from six slices) from a different rat was removed from the membrane insert and soaked in artificial cerebrospinal fluid (aCSF; with composition of 124 mM NaCl, 26 mM NaHCO_3_, and 10 mM glucose; 3 mM KCl, 2 mM CaCl_2_, and 1 mM MgCl_2_; 10 mM HEPES, pH 7.4).

The slice was placed on the center of the MEA and the surrounding solution was removed using a pipette. Any remaining solution between the slice and the MEA surface was removed using tissue paper to minimize contact. Fresh aCSF solution was then quickly introduced on top of the slice. The hippocampal slice on the array was submerged completely in aCSF solution bubbled at 3 mL/min and stabilized for 1 h at 33°C. The slice and MEA array were then transferred into an MEA1060 amplifier interface. The solution in the array was grounded using an Ag/AgCl pellet. After each experiment, the array was cleaned with 2% ultrasonol 7 (Carl Roth GmbH) in distilled water for 30 min, rinsed with distilled water, and then kept in distilled water at room temperature.

### 2.4. Induction of LTP for Hippocampal Slice Electrophysiology

**Figure 2:**
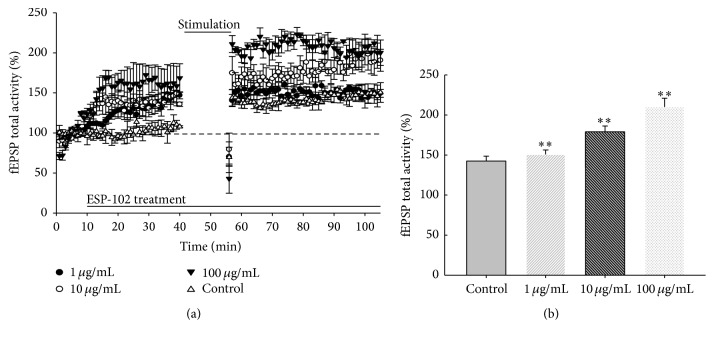
Effects of ESP-102 on long-term potentiation (LTP) in rat hippocampal tissue (*n* = 3–5/group). (a) Grouped data showing time course of LTP from all recordings made from control or ESP-102 treated (1 *μ*g/mL, 10 *μ*g/mL, and 100 *μ*g/mL) hippocampal tissue. (b) Average LTP amplitude measured 30–40 min after HFS. ^*∗∗*^ *p* < 0.01 versus control group.

Single slices from multiple animals (*n* = 3–5) were used for each experimental group. A bipolar stimulation was applied to the stratum radiatum of the CA2 region to stimulate Schaffer collateral (SC) and commissural pathways. Baseline synaptic responses were evoked by stimulation at 0.033 Hz (180 *μ*s pulse-width) and recorded for 40 min. For all experiments, test stimuli were delivered once a minute, and the stimulus intensity was set to give a baseline field EPSP 50% of maximum. High-frequency stimulation (HFS) consisted of a 1 s train of pulses delivered at 100 Hz, with a total of 100 pulses. One set of HFS stimulation consisted of 3 trains with a 3 min intertrain interval (Figure 1(b)). The HFS protocol was more successful than the conventional TBS stimulation. Moreover, this stimulus protocol was set up to not reach functional saturation of LTP with enough headroom (Figure 2). We strictly avoided using more than one HFS-induced condition because further stimulation could lead to an increment of LTP [13, 14]. Field excitatory postsynaptic potentials (fEPSPs) were recorded in whole sites of the hippocampus. During experiments, the slices were continuously perfused with fresh aCSF solution (bubbled with 95% O_2_, 5% CO_2_) at a rate of 3 mL/min.

### 2.5. Induction of Long-Term Depression (LTD) for Hippocampal Slice Electrophysiology

Single slices from multiple animals (*n* = 3–6) were used for each experimental group. Bipolar electrical stimulation was applied to the stratum radiatum of CA2 to stimulate the SC/commissural pathway. The intensity of bipolar test pulse (or baseline) stimulation was set at 100 mA; this value was optimized to provide 40–65% of the maximum tissue response and was delivered once every 60 seconds. Baseline responses were evoked for at least 30 min, of which the last 10 minutes was recorded, before the low-frequency conditioning stimulation (1 Hz for 15 minutes; 900 total pulses; Figure 1) was applied to induce LTD. After the conditioning stimulation, fEPSPs were recorded every 60 sec for another 75 min by 59 microelectrodes spanning the hippocampus.

### 2.6. Electrophysiology Data Processing

MC_Rack (v.3.2.1.0, Multi Channel Systems) was used to digitize the analog MEA signal and isolate EPSPs from triggering amplitudes greater than 40 mV, and a custom MATLAB (v.7.0.1, Mathworks, Inc.) program was used to remove stimulus artifacts and integrate the evoked field potential trajectory, as reported previously [6, 11].

### 2.7. Western Blot Analysis

For western blot experiments we used hippocampal tissue that was incubated for 24 h with culture medium (control), ESP-102 (10 *μ*g/mL), or ESP-102 with scopolamine (10 mM) (18 tissue samples, *n* = 3). Tissue slices were used for electrophysiology experiments after 14 days of organotypic hippocampal slice culture. Briefly, previously sectioned hippocampus was removed from storage at −80°C and the region of interest was dissected on dry ice. Dissected regions were homogenized by sonication in cold cell lysis buffer containing phosphatase inhibitors and a complete protease inhibitor cocktail. Hippocampus extracts were then incubated on ice for 30 min and centrifuged at 14,000 ×g for 10 min at 4°C. Protein concentrations of supernatants were measured using Bradford protein assay and equal amounts of protein were separated on 10% SDS-PAGE gels and transferred to PVDF membranes. The membranes were blocked in TBS with 0.1% Tween 20 containing 5% dry skim milk for 1 h and incubated in 5% skim milk with primary antibodies overnight at 4°C. Antibodies used were polyclonal antibody against brain-derived neurotrophic factor (BDNF) (sc-33904, Santa Cruz Biotechnology, Santa Cruz, CA, USA), polyclonal antibody against glutamate receptor-2 (GluR-2) (sc-7610, Santa Cruz Biotechnology), and mouse monoclonal antibody against beta-actin (sc-47778, Santa Cruz Biotechnology). Membranes were washed and further incubated for 1 h at room temperature with secondary antibodies (goat anti-mouse and donkey anti-goat IgG conjugated to horseradish peroxidase; sc-2005 and sc-2056, Santa Cruz Biotechnology). After a final wash, the bands were developed using a horseradish peroxidase-conjugated secondary antibody and visualized with an ECL Western Blotting Detection System (ATTO system). All experiments were repeated at least three times with different batches of tissue samples and the results were fully reproducible.

### 2.8. Statistical Analysis

The results are expressed as the mean ± standard error of the mean (SEM). Statistical comparisons were conducted using one-way analysis of variance (ANOVA) followed by Duncan's* post hoc* multiple comparison test using SPSS 20.0 for windows (SPSS Inc., Chicago, IL, USA). Differences with *p* < 0.01 or *p* < 0.001 were determined using two-tailed Student's *t*-test. The changes in western blot bands were compared among the groups using one-way ANOVA.

## 3. Results

### 3.1. ESP-102 Induced LTP in Organotypic Hippocampal Slices in a Dose-Dependent Manner

**Figure 3:**
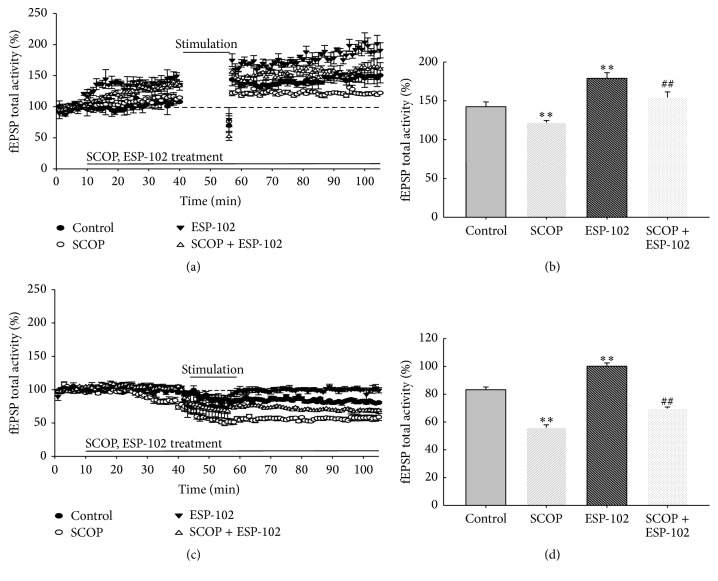
Effects of ESP-102 on long-term potentiation (LTP) and long-term depression (LTD) in hippocampal tissue of rats treated with scopolamine (*n* = 3–6/group). (a) Grouped data showing time course of LTP from all recordings made from hippocampal tissue treated with ESP-102 (10 *μ*g/mL), scopolamine (10 *μ*M), and scopolamine + ESP-102. (b) Average LTP amplitude measured 30–40 min after HFS. (c) Grouped data showing time course of LTD from all recordings made from hippocampal tissue treated with ESP-102 (10 *μ*g/mL), scopolamine (10 *μ*M), and scopolamine + ESP-102. (d) Average LTD amplitude measured 30–40 min after HFS. ^*∗∗*^ *p* < 0.0 versus control group. ^##^ *p* < 0.01 versus scopolamine group.

Previously, our colleagues investigated the alleviating effect of ESP-102 on memory impairment in a rat model and neuroprotection against toxic substances with regard to synaptic plasticity in* in vitro* cell cultures [1–3]. In the present study, we examined changes in LTP and LTD with ESP-102 treatment. In Figure 2, the field excitatory postsynaptic potential (fEPSP) in the control group without prior LTP induction was set to 100%. The ESP-102 treated group was set to 150% with LTP induction and the fEPSP increased in a concentration-dependent manner. Changes in LTP and LTD were repeatedly observed with ESP-102 treatment (Figure 3).

### 3.2. The Mechanism of Action of ESP-102 in Synaptic Plasticity

**Figure 4:**
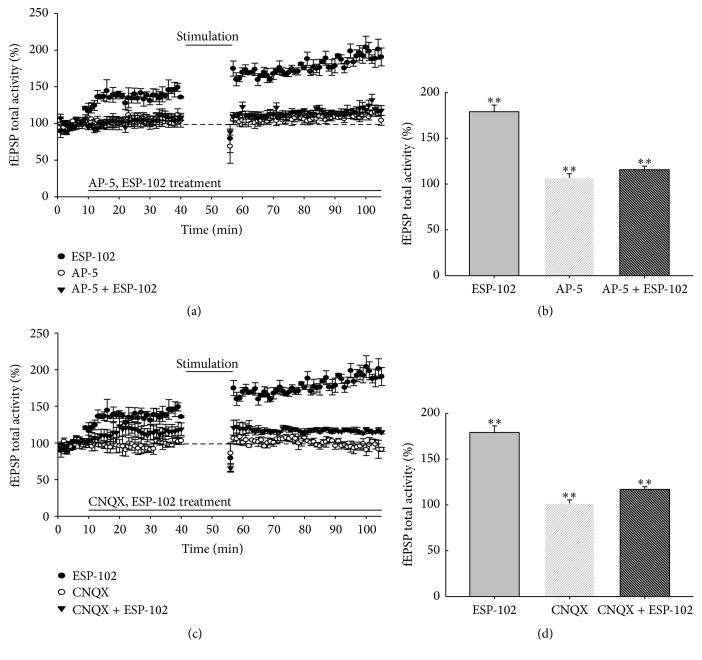
Effects of ESP-102 and antagonists AP-5 and CNQX on long-term potentiation (LTP) in rat hippocampal tissue (*n* = 3–5/group). (a) Grouped data showing time course of LTP from all recordings made from hippocampal tissue treated with ESP-102 (10 *μ*g/mL), AP5 (10 *μ*M), and AP5 + ESP-102. (b, d) Average LTP amplitude measured 30–40 min after HFS. (c) Grouped data showing time course of LTP from all recordings made from hippocampal tissue treated with ESP-102 (10 *μ*g/mL), CNQX (10 *μ*M), and CNQX + ESP-102. Means with different letters differ at *p* < 0.01 (*∗∗*) level by ANOVA and Duncan's multiple range test and two-tailed Student's *t*-test.

To identify the mechanism by which ESP-102 affects neural plasticity, we compared the EPSP activity in the presence of NMDA and AMPA/kainate receptor antagonists. The antagonistic activity of AP-5 and CNQX was virtually unaffected by ESP-102 treatment (Figure 4). Therefore, it seems likely that ESP-102 exerts its effects at the presynaptic region through increasing neurotransmitter release.

### 3.3. Neuroprotective Effect of ESP-102 by Restoring Neurotrophic Factors

We evaluated levels of neurotrophic factors such as brain-derived neurotrophic factor (BDNF) and glutamate receptor-2 (GluR-2) in the hippocampus before and after ESP-102 treatment because they seem to be critical cues in hippocampal development and neurogenesis. BDNF may play vital roles in promoting neuronal growth as well as survival during development. It seems to be also crucial for regulating the integrity and function of neurons throughout entire lifetime [14].

**Figure 5:**
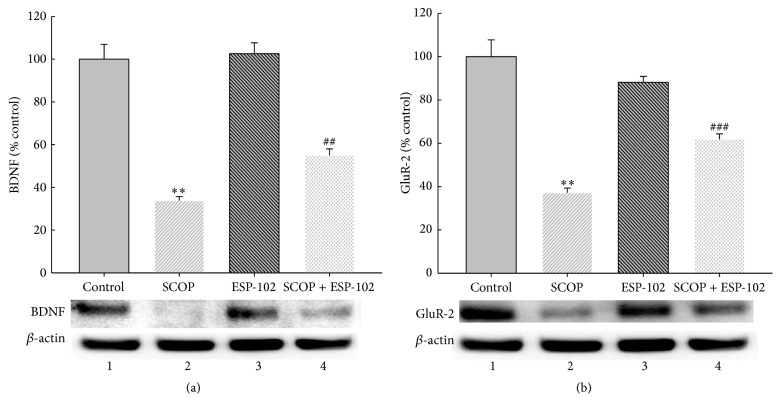
Effects of ESP-102 on the protein expression of brain-derived neurotrophic factor (BDNF) and glutamate receptor-2 (GluR-2) in rats with scopolamine-induced hippocampal impairment by western blot analysis (*n* = 4/group). (a) Protein levels of BDNF in rat hippocampus were measured by western blot analysis using anti-BDNF specific antibody. *β*-actin levels were assessed to confirm equal protein loading. Quantitative analysis of relative band intensity for BDNF/*β*-actin is represented in the graph. (b) Protein levels of GluR-2 in rat hippocampus were measured using anti-GluR-2 specific antibody. Quantitative analysis of relative band intensity for GluR-2/*β*-actin is represented in the graph. Band 1: control group; Band 2: scopolamine; Band 3: ESP-102; Band 4: scopolamine and ESP-102. The data were normalized against *β*-actin levels and expressed as percentage of control values. ^*∗∗*^ *p* < 0.01 versus control group. ^##^ *p* < 0.01, ^###^ *p* < 0.001 versus scopolamine group.

As shown in Figure 5, treatment with scopolamine resulted in a significant decrease in expression levels of BDNF and GluR-2 in the rat hippocampus compared to the control group (*p* < 0.01) as determined by western blotting. ESP-102 alone did not affect BDNF and GluR-2 levels; however, ESP-102 reversed the scopolamine-induced reduction of BDNF expression (Figure 5(a), *p* < 0.01) and dramatically increased GluR-2 expression (Figure 5(b), *p* < 0.001).

## 4. Discussion

In spite of the decades of research for dementia treatment, the complete understanding of the pathophysiology has not been yet reached, not to mention promising therapy. The most common form of dementia is Alzheimer's disease, also known as age-related dementia [15–17]. Because Alzheimer's disease mainly affects the increasing population of elderly individuals, there is a great demand for better drugs with fewer side effects. For this purpose it is logical to develop novel therapeutic candidates from traditional herbal medicines and botanical drugs with already proven efficacy and safety [6]. Based on previous findings of the neuroprotective activity of ESP-102, it was presumed that it might affect hippocampal neural plasticity [11]. Therefore, we examined the effects of ESP-102 on the conditions of LTP and LTD in organotypic hippocampal slice cultures from 7-day-old Sprague-Dawley rats.

Our results show that treatment with ESP-102 increased the overall neuronal plasticity as well as neuroprotective effect. Treatment with ESP-102 alone increased LTP in a dose-dependent manner (Figure 2). Combined treatment with NMDA and AMPA/kainate receptor antagonists in presence of ESP-102 suggested that ESP-102 seemed to be an effector of neuronal plasticity via glutamatergic synapses. Previously, BDNF is known for role in associative learning and countereffect of LTP in presynaptic region [14, 18, 19]. Somehow, our experiments with glutamatergic antagonists and protein expression level analyses did not provide precise information on pre- or postsynaptic involvement. Nevertheless, the recovery of scopolamine-suppressed BDNF and GluR-2 protein levels upon ESP-102 treatment seemed to suggest unnoticeable involvement in both presynapse and postsynapse regions. For these reasons the new findings for ESP-102 function in cerebral neurons emphasize its potential role in neuronal revitalization, even in neurodegenerative conditions. In the near future, we hope to investigate the target molecules in neurons and the subsequent intracellular signaling through ESP-102. In addition, it would be very challenging work to examine systemic modification of neuronal network after injection of ESP-102 in animal models for neurodegenerative diseases.
